# The added value of free preparatory activities for widening access to medical education: a multi-cohort study

**DOI:** 10.1186/s12909-023-04191-7

**Published:** 2023-03-29

**Authors:** S. Fikrat-Wevers, W. E. De Leng, W. W. Van Den Broek, A. M. Woltman, K. M. Stegers-Jager

**Affiliations:** Institute of Medical Education Research Rotterdam, Erasmus MC, University Medical Center Rotterdam, Room AE-241, PO Box 2040, 3000 CA Rotterdam, the Netherlands

**Keywords:** Diversity, Widening access, Medical school selection, Preparatory activities, Free resources, Transition (Three to ten keywords representing the main content of the article)

## Abstract

**Background:**

Medical schools are reported to be less accessible to students with non-traditional backgrounds. These students face barriers when applying for and transitioning to medical school, which may be reduced by offering free preparatory activities. By equalizing access to resources, these activities are expected to reduce disparities in selection outcomes and early academic performance. In the present study, four free institutionally-provided preparatory activities were evaluated by comparing the demographic composition of participating and non-participating applicants. Additionally, the association between participation and selection outcomes and early academic performance was investigated for subgroups (based on sex, migration background and parental education).

**Methods:**

Participants were applicants to a Dutch medical school in 2016-2019 (*N* = 3592). Free preparatory activities included Summer School (*N* = 595), Coaching Day (*N* = 1794), Pre-Academic Program (*N* = 217), and Junior Med School (*N* = 81), supplemented with data on participation in commercial coaching (*N* = 65). Demographic compositions of participants and non-participants were compared using chi-squared tests. Regression analyses were performed to compare selection outcomes (curriculum vitae [CV], selection test score, probability of enrolment) and early academic performance (first-course grade) between participants and non-participants of demographic subgroups, controlling for pre-university grades and participation in other activities.

**Results:**

Generally, no differences in sociodemographic compositions of participants and non-participants were found, but males participated less often in Summer School and Coaching Day. Applicants with a non-Western background participated less often in commercial coaching, but the overall participation rate was low and participation had negligible effects on selection outcomes. Participation in Summer School and Coaching Day were stronger related with selection outcomes. In some cases, this association was even stronger for males and candidates with a migration background. After controlling for pre-university grades, none of the preparatory activities were positively associated with early academic performance.

**Conclusions:**

Free institutionally-provided preparatory activities may contribute to student diversity in medical education, because usage was similar across sociodemographic subgroups, and participation was positively associated with selection outcomes of underrepresented and non-traditional students. However, since participation was not associated with early academic performance, adjustments to activities and/or curricula are needed to ensure inclusion and retention after selection.

**Supplementary Information:**

The online version contains supplementary material available at 10.1186/s12909-023-04191-7.

## Background

A diverse student population is important for promoting excellence in medical education, and for equalizing access to high quality health care [1, 2]. Nevertheless, medical schools are reported to be less accessible to students with non-traditional backgrounds, defined as those with lower socioeconomic status (SES), and from ethnic minority groups [3–5]. One of the reasons for this is that students with non-traditional backgrounds generally have less access to resources to successfully prepare for the selection for and the transition to medical school [6]. Therefore, researchers have suggested that more attention should be paid to increasing student diversity by intervening during the preparation phase [7–10]. The present study evaluates whether applicants from different demographic groups are represented in the participants of freely accessible preparatory activities, and whether participation in such activities is associated with selection outcomes and subsequent early academic performance for demographic subgroups.

One potential threat to student diversity in medical education is that students with non-traditional backgrounds have less access to resources, which causes them to perceive more barriers compared to traditional students when applying for medical school [9, 11, 12]. Some of those barriers relate to the selection procedure, such as a lack of knowledge about the selection procedure and reduced possibilities to conduct extracurricular activities [9, 12, 13]. An additional potential barrier is related to the growing number of commercial agencies providing coaching activities to prepare applicants for the selection procedure [14]. Due to their financial costs, these activities are less accessible to applicants with lower SES, potentially increasing inequity in admission chances [15–17]. Other barriers for applicants with non-traditional backgrounds are more related to the transition to medical school in general, such as a fear of not fitting in because of their background and a lack of confidence in their ability to stay in the course [11, 18].

One suggested approach to reduce aforementioned barriers for applicants with non-traditional backgrounds, is organizing free preparatory activities for the selection procedure [7, 8, 19]. Such activities may equip these applicants with the knowledge they need and increase their confidence in gaining successful admission. Moreover, making preparatory activities accessible to all prospective students can result in more equal starting positions to the selection procedure for applicants from different backgrounds [8, 16]. Nevertheless, other factors than financial barriers could affect non-traditional applicants’ awareness of and time to participate in preparatory activities, such as work and family obligations [15]. Consequently, it is relevant to investigate the usage of such activities for different subgroups. There is limited evidence about whether free preparatory activities succeed in attracting a diverse group of applicants, and results thus far are mixed [6, 16, 20].

Preparatory activities can have different goals, such as improving applicants’ selection outcomes and preparing prospective students for their transition into medical school. Many commercial agencies provide activities that focus on increasing selection chances, although they are not always effective [16, 21–25], and selection outcomes of commercially-coached students can overpredict their future academic performance [16, 26]. Research on free institutionally-provided activities for improving selection outcomes is limited. One study focusing on free coaching activities found a positive effect of participation on selection outcomes, but no association between participation and subsequent academic performance [16]. This suggests that these activities did better prepare applicants for selection, but did not improve the underlying skills necessary for medical school. Other research found a positive association between the use of free online provided practice tests and selection outcomes, while attendance at free preparation courses provided by prior educational institutions was negatively associated with selection outcomes [24, 25].

With respect to activities focusing on the transition into medical education, researchers have mainly concentrated on interventions specifically targeting prospective students with non-traditional backgrounds [27, 28]. Such interventions are extensive and resource intensive, while their effectiveness with respect to increasing academic performance has been shown to be limited [29, 30]. The present study focuses on a variety of activities, ranging in extensiveness, most of them available to all prospective students. One of those activities, a pre-academic transition program, was found to be effective in increasing early academic performance in other disciplines (e.g., law) [20], but has not yet been investigated within the field of medical education. Another one of those activities, a scientific pre-university program, was found to increase interest in an academic career for medical students [31], but its usage by diverse subgroups of prospective students is yet unknown.

To date, no studies have reported on the differential effectiveness – in terms of increasing selection outcomes and early academic performance – of free preparatory activities for subgroups of (prospective) students. Such information is relevant, since these initiatives are aimed at equalizing access to medical education. Drawing on the work of Bourdieu [32], we expect that free preparatory activities can reduce disparities in selection outcomes and academic performance. The provision of free preparatory activities may buffer the lack of social capital (i.e., resources accessible through personal connections) which students with non-traditional backgrounds more often experience [32]. Additionally, such activities may make the implicit rules and practices of the academic culture that are associated with the dominant group more explicit to students with non-traditional backgrounds (i.e., cultural capital) [32]. Understanding the impact of these activities on subgroups of students with non-traditional backgrounds can help medical schools in increasing student diversity. In the Netherlands, the context of the present study, male students are underrepresented [33], and students with a migration background and first-generation students are considered students with non-traditional backgrounds [34].

Thus, there is a lack of research into the usage of free institutionally-provided preparatory activities by different demographic subgroups, and their differential outcomes for these subgroups. With the present study, we aimed to answer the following research questions: (1) How do the demographic compositions of participating and non-participating applicants compare in various free institutionally-provided preparatory activities (varying in goal, timing and intensity), as well as in commercial coaching activities for the selection of an undergraduate medical program? (2) Does participation in different free institutionally-provided preparatory activities predict selection outcomes and does this relationship differ for subgroups? And (3) Does participation in different free institutionally-provided preparatory activities predict early academic performance and does this relationship differ for subgroups? Subgroups were based on background variables that are associated with selection and early academic performance: sex, migration background (as an indicator of ethnicity) and parental education (as an indicator of SES) [3, 5, 33–37].

## Methods

### Design

In this retrospective multi-cohort study, we compared the demographic composition, selection outcomes, and early academic performance of applicants who participated and those who did not participate in four free preparatory activities provided by one medical school.

### Context

The present study was conducted at the Erasmus MC Medical School, Rotterdam, the Netherlands. In the Netherlands, applicants apply to their program of choice at one specific institution. Each year, applicants can only apply to one medical program. By law, institutions are required to include at least two selection criteria, but programs are responsible for designing their own selection procedure. Therefore, the selection procedures and accompanied free preparatory activities are specifically designed for the applicants’ program of choice at that specific institution.

The selection procedure for the undergraduate program of Erasmus MC medical school consists of three equally-weighted components: i) Grade point average of pre-final year (Year 5) of pre-university education (year 5 pu-GPA), ii) a score based on the extracurricular activities undertaken by candidates (curriculum vitae; CV-score) and iii) a score on multiple cognitively-based tests (selection test score). These three components are standardized and averaged to attain the final score on which selection is based. Over the years, small adaptations have been made within the components of the selection procedure. For cohorts 2016-2018, candidates with a year 5 pu-GPA of ≥7.5 out of 10 were directly admitted (without assessment of the other two components). For cohort 2019, this benchmark was raised to a year 5 pu-GPA of ≥8.0.

### Participants and procedure

**Fig. 1:**
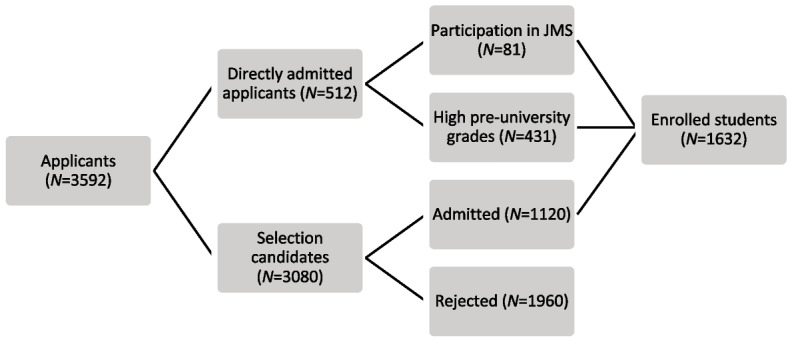
Flow chart of possible admission pathways, including descriptive statistics of applicants for cohorts 2016-2019 JMS = Junior Med School

The study sample comprised of (prospective) students who applied to the undergraduate medical program for the cohorts 2016-2019, referred to as applicants. Figure 1 provides an overview of the different admission pathways. A distinction was made between selection candidates who took part in the selection procedure and directly admitted applicants (either based on year 5 pu-GPA or participation in the Junior Med School). Enrolled students included admitted selection candidates and directly admitted applicants who were enrolled in the first year of the program. Admitted applicants generally all enroll in the medical program. Data on selection outcomes of selection candidates, early academic performance of enrolled students, participation in institutionally-provided preparatory activities and sex were retrieved from the university administration system. Data on participation in commercial coaching and additional demographics (i.e., parental education, migration background) were inquired using a survey administered during the selection procedure. For enrolled students who had not filled out the demographics survey, information regarding migration background was obtained through a Dutch database of students in higher education (i.e., 1 Cijfer HO). Informed consent was provided for the demographics survey. Applicants were informed that their answers on the demographics survey would not influence selection outcomes, and that the researchers operated independently from the selection committee. The Medical Ethics Committee Erasmus MC of Rotterdam, the Netherlands declared the study exempt from review.

### Preparatory activities

**Fig. 2:**
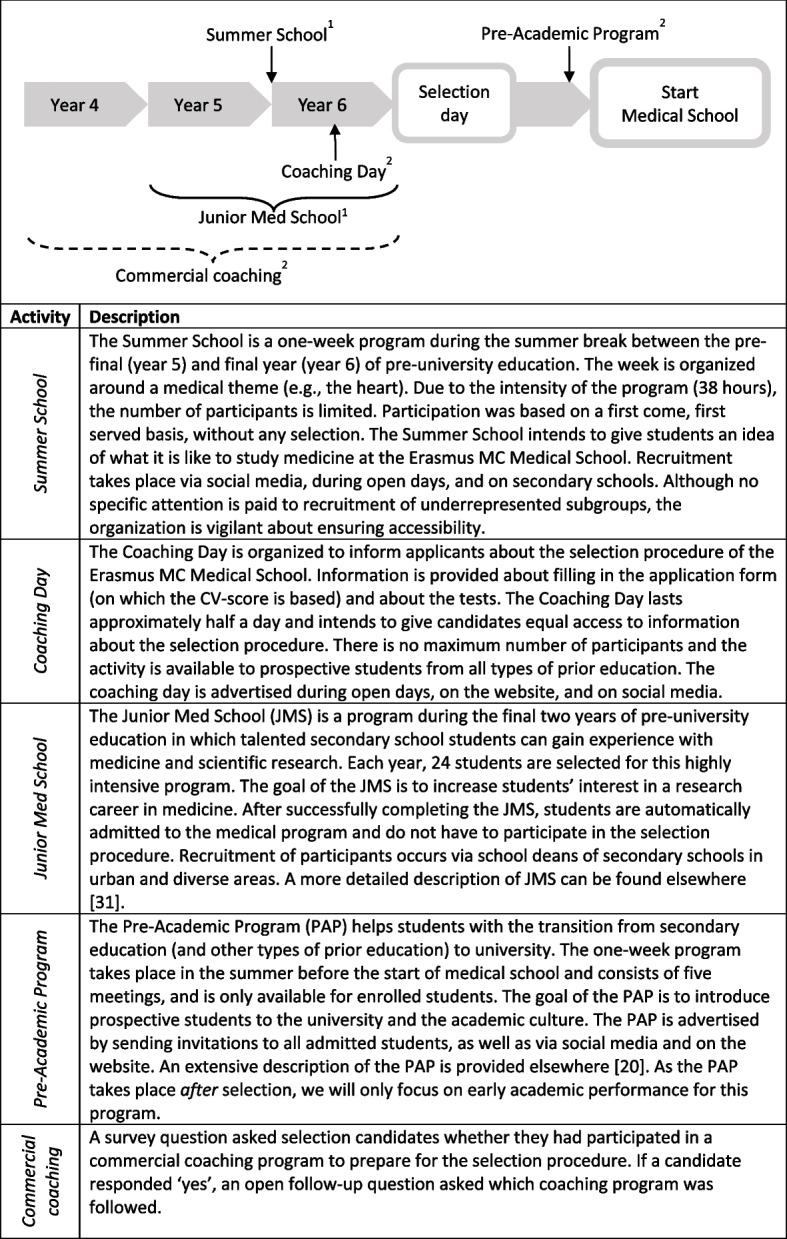
Chronological overview and description of preparatory activities ^1^Only accessible to applicants from pre-university education.^2^Accessible for applicants regardless of prior education

We used data on participation in four institutionally-provided activities and commercial coaching. Figure 2 provides a chronological overview and description of all activities, distinguishing between activities only available for applicants from secondary school at a pre-university educational level, and activities available for applicants with any form of prior education. In the Netherlands, the vast majority of applicants comes from pre-university education, but alternative forms of prior education are also allowed, such as another university program or higher vocational education.

### Student characteristics

The following student characteristics were included: sex (female or male), migration background, and parental education. Migration background was determined in accordance with Statistics Netherlands (CBS) by the country of birth of the students’ parents, distinguishing between no migration background, a Western migration background and a non-Western migration background. All countries in Europe (excluding Turkey), in North America, in Oceania, and Indonesia and Japan were considered Western. Non-Western countries included all countries in Africa, Asia (excluding Indonesia and Japan), Latin America and Turkey. This taxonomy is largely based on geographical origin, but also on socioeconomic and sociocultural position. Parental education was determined by the educational level of the students’ parents. When neither parent had attended higher education, a student was classified as a first-generation university student.

### Control variables

Pu-GPA is known to be associated with both selection outcomes and academic performance [5, 36]. Therefore, pu-GPA (on a scale of 1-10) was included as a control variable. Since pu-GPA was not available for selection candidates with non-standard prior education, a categorical variable was added to the analyses to indicate whether pu-GPA was available (yes/no). Missing values for pu-GPA were substituted with the mean pu-GPA. Year 5 pu-GPA was used as a control variable for selection outcomes, while year 6 pu-GPA was used as a control variable for early academic performance. The use of pu-GPA of both year 5 and year 6 yielded the most complete information for selection outcomes and early academic performance, respectively: year 5 pu-GPA was available for both admitted and rejected selection candidates (but we did not have access to this data for directly admitted applicants), while year 6 pu-GPA was available for the majority of the enrolled students, including directly admitted applicants (but not for rejected selection candidates).

### Outcome variables

With respect to selection outcomes, two variables were included: CV-scores and selection test scores. For CV-scores and selection test scores, the selection committee calculated raw scores based on their scoring rubrics, which were transformed into standardized *Z*-scores for the final ranking of candidates. These *Z*-scores were made available to the researchers and used for the analyses. Regarding early academic performance, we included the first course grade of the first year, which consists of the weighted average score on two exams. The theme of this first course was ‘The healthy human being’, and included an introduction on numerous biomedical subjects, such as cell biology, anatomy, and neurology. Both exams covered the subjects of the first course. The first exam was administered one-and-a-half months after the start of the medical program, and the second after two-and-a-half months. First course grade was selected as an outcome measure, because we were interested in whether participation prepared students better for the *transition* into higher education. Moreover, academic performance during the first months at university is predictive of subsequent performance [38, 39].

### Analyses

Demographic compositions of participating and non-participating selection candidates in each preparatory activity were compared using chi-squared tests (research question 1). Cramer’s *V* was used to calculate the effect sizes of the chi-squared tests. The guidelines for interpretation of *V* depend on the degrees of freedom [40]. The group of non-participants is composed of selection candidates who had not participated in the activity under consideration. The demographic composition of each group was described for the variables sex, migration background and parental education. Independent samples *t*-tests were conducted to investigate whether year 5 pu-GPA differed statistically significantly between participants and non-participants.

Associations between participation in preparatory activities and selection outcomes were examined using linear regression analyses (research question 2). Only selection candidates were included in this analysis. Independent factors included participation in Coaching Day, Summer School, and commercial coaching. The outcome variables included *Z*-scores on CV and *Z*-scores on selection tests. In Model 1, the associations between participation and selection outcomes were examined separately for each activity, while in Model 2, all activities were included simultaneously to control for participation in other activities. In Model 3, we additionally controlled for year 5 pu-GPA. In addition, logistic regression analyses were performed to calculate odds ratios (*OR*) for the association between participation and the probability of enrolment in the program, using the same model structure. An OR of > 1 indicates an increased likelihood of enrolment.

Linear regression analyses were also used to estimate associations between participation and early academic performance (research question 3). All enrolled students were included in this analysis. This time, independent factors included participation in Summer School, Coaching Day, JMS, PAP, and commercial coaching. The outcome variable included first course grade on a scale from 1 to 10, with 10 as the highest grade and 5.5 as passing grade. The same model structure was used as for selection outcomes, but with year 6 pu-GPA included in Model 3. For JMS, we could only control for participation in PAP and year 6 pu-GPA, because none of the JMS participants participated in any of the other activities.

In order to investigate subgroup differences in associations between participation and selection outcomes (research question 2), and between participation and early academic performance (research question 3), main effects of background variables (sex, migration background, and parental education) and interaction terms (participation*sex, participation*migration background, participation*parental education) were included in Model 3 for all outcome measures. Interaction effects were only investigated for free institutionally-provided preparatory activities.

Analyses were executed using SPSS version 26. For all statistical analyses, assumptions were checked in accordance with the guidelines of Field [41], and all assumptions were met. For the *t*-tests, Cohen’s *d* was for distinguishing small (*d* > .20), medium (d > .50), and large (d > .80) effects. For the linear regression analyses, unstandardized regression coefficients (*B*) were used, and *R*^2^ was used to interpret the effect size based on the guidelines of Cohen, with *R*^2^ = 0.02, *R*^2^ = 0.13 and *R*^2^ = 0.26 respectively indicating small, medium and large effects [42]. We interpreted *OR* of > 1.68 as a small effect, *OR* of > 3.47 as a medium effect, and *OR* of > 6.71 as a large effect [43].

## Results

### Descriptive statistics

In total, 3592 applicants (68.9% female, mean age 19.2 years) were registered in the database (Fig. 1). Three-thousand-and-eighty of them were candidates in the selection procedure, whilst the other 512 were directly admitted either based on participation in JMS (*N* = 81) or pre-university grades (*N* = 431). More than half of the subjects (*N* = 2086) participated in at least one of the preparatory activities, and 1632 were enrolled as a student in the first year of medical school. Data on migration background were available for 3072 subjects (selection candidates who filled out the demographics questionnaire and enrolled students) of whom 61.9% had no migration background, 28.0% had a non-Western migration background and 10.1% had a Western migration background. Data on parental education were only present for selection candidates who filled out the demographics questionnaire, resulting in 2523 individuals, of whom 28.0% were classified as first-generation university students.

### Research question 1: demographic composition of participants in preparatory activities

Demographic characteristics of participating and non-participating selection candidates in each preparatory activity are presented in Table 1. The same information for enrolled students can be found in Additional file 1. It is noteworthy that the number of participants in most activities was relatively small, with the exception of the Coaching Day. This is due to the fact that two activities had a maximum number of participants (Summer School and JMS), and that the PAP was only accessible to admitted applicants.

**Table 1:** Demographic characteristics of participating and non-participating selection candidates in each preparatory activity

		Selection candidates	Summer School participant		Coaching Day participant		JMS participant^**b**^		PAP participant^**c**^		Commercial coaching	
			Yes	No	Yes	No	Yes	No	Yes	No	Yes	No
		*N (%)*	*N (%)*	*N (%)*	*N (%)*	*N (%)*	*N (%)*	*N (%)*	*N (%)*	*N (%)*	*N (%)*	*N (%)*
	Total	3080	550	2530	1637	1443	81	3080	210	1330	65	2405
**Sex**	Male	965 (31.3)	142 (**25.8**)	823 (**32.5**)	442 (**27.0**)	523 (**36.2**)	27 (33.3)	965 (31.3)	57 (27.9)	387 (29.1)	23 (35.4)	743 (30.9)
	Female	2115 (68.7)	408 (**74.2**)	1707 (**67.5**)	1195 (**73.0**)	920 (**63.8**)	54 (66.7)	2115 (68.7)	153 (72.1)	943 (70.9)	42 (64.6)	1662 (69.1)
	*Chi-squared*		**χ2 = 9.46,** ***df*** **= 1,** ***p*** **= .002**		**χ2 = 30.46,** ***df*** **= 1,** ***p*** **< .001**		χ2 = .15 *df* = 1, *p* = .70		χ2 = 0.34, *df* = 1, ***p*** = .56		χ2 = .60, *df* = 1, ***p*** = .44	
**Migration background**	No	1782 (62.2)	362 (**68.3**)	1420 (**60.8**)	1003 (63.4)	779 (60.6)	52 (68.4)	1782 (62.2)	127 (**60.5**)	945 (**71.1**)	49 (**76.6**)	1449 (**60.7**)
	Western	279 (9.7)	32 (**6.0**)	247 (**10.6**)	144 (9.1)	135 (10.5)	6 (7.9)	279 (9.7)	16 (**7.6**)	125 (**9.4**)	7 (**10.9**)	233 (**9.8**)
	Non-western	806 (28.1)	136 (**25.7**)	670 (**28.7**)	435 (27.5)	371 (28.9)	18 (23.7)	806 (28.1)	67 (**31.9**)	260 (**19.5**)	8 (**12.5**)	706 (**29.6**)
	*Chi-squared*		**χ2 = 14.46,** ***df*** **= 2,** ***p*** **= .001**		χ2 = 2.79, *df* = 2, ***p*** = .25		χ2 = 1.24, *df* = 2, ***p*** = .54		**χ2 = 16.58,** ***df*** **= 2,** ***p*** **< .001**		**χ2 = 8.90,** ***df*** **= 2,** ***p*** **= .01**	
**Parental education**	No 1st gen	1776 (71.9)	324 (71.8)	1452 (71.9)	996 (72.0)	780 (71.7)	n.a.	1776 (71.9)	108 (71.5)	758 (77.3)	48 (73.8)	1727 (71.8)
	1st gen	695 (28.1)	127 (28.2)	568 (28.1)	387 (28.0)	308 (28.3)	n.a.	695 (28.1)	43 (28.5)	223 (22.7)	17 (26.2)	678 (28.2)
	*Chi-squared*		χ2 = .00, *df* = 1, ***p*** = .99		χ2 = .03, *df* = 1, ***p*** = .86		n.a.		χ2 = 2.40, *df* = 1, ***p*** = .12		χ2 = .13, *df* = 1, ***p*** = .72	
**Year 5 pu-GPA**^a^			*M (SD)*	*M (SD)*	*M (SD)*	*M (SD)*	*M (SD)*	*M (SD)*	*M (SD)*	*M (SD)*	*M (SD)*	*M (SD)*
		1773 (57.6)	6.82 (0.55)	6.77 (0.57)	**6.82** (0.54)	**6.73** (0.59)	n.a.	6.78 (0.56)	**7.20** (0.45)	**7.10** (0.49)	6.83 (0.55)	6.74 (0.53)
	*t-test*		*t* = −1.61, *df* = 1747, *p* = .11		***t*** **= 3.16,** ***df*** **= 1189,** ***p*** **= .002**		n.a.		***t*** **= 1.98,** ***df*** **= 831,** ***p*** **= .048**		*t* = −1.18, *df* = 1577, *p* = .24	

The chi-square test compares the prevalence of each category (e.g., males) of candidates participating in a free preparatory activity with the same category (e.g., males) of candidates not participating in that free preparatory activity. Bold indicates a statistically significant effect (*p* < .05)

*JMS* Junior Med School, *PAP* Pre-Academic Program, *N* number of individuals, *1st gen* first-generation university student, *pu-GPA* pre-university grade point average, *n.a.* not available, *M* mean, *SD* standard deviation, *df* degrees of freedom

^a^ pu-GPA is not accessible for students with non-standard prior education and JMS participants. ^b^for JMS, the participant group consists of directly-admitted applicants, while the non-participant group consists of selection candidates ^c^for PAP, the non-participant group consists of selection candidates enrolled in the first year of medical school, because this activity was only available for admitted students

In total, 595 selection candidates participated in Summer School. Females were overrepresented in the group of Summer School participants, in comparison to the group of non-participants (74.3% vs. 67.8%, χ^*2*^(1) = 9.46, *p* = .002, *V* = .05, small effect; Table 1). Additionally, a statistically significant difference in migration background was observed between participants and non-participants in the Summer School (χ^*2*^(2) = 14.46, *p* = .001, *V* = .07, small effect). The standardized residuals indicated that this statistically significant difference was mainly caused by an underrepresentation of students with a Western migration background (*z*_res_ = − 2.7) and an overrepresentation of students without a migration background (*z*_re*s*_ = 1.8). Participants did not statistically significantly differ from non-participants based on parental education and year 5 pu-GPA.

The Coaching Day was the most attended institutionally-provided activity (*N* = 1794; Table 1). The group of Coaching Day participants also consisted of statistically significantly more females than the group of non-participating selection candidates (73.2% vs. 64.6%, χ^*2*^(1) = 30.46, *p* < .001, *V* = 0.09, small effect). Coaching Day participants and non-participants did not statistically significantly differ in terms of migration background and parental education, but participants had a statiscially significantly higher year 5 pu-GPA than non-participants (*t* = 3.16, *df* = 1189, *p* = .002, *d* = 0.16, negligible effect).

Participants in the JMS (*N* = 81), the only selective institutionally-provided activity, did not statistically significantly differ from non-participants in their sex and migration background. Data on parental education and year 5 pu-GPA were not available for JMS-participants.

For PAP (*N* = 217), we found a statistically significant difference in migration background between participants and non-participants (χ^*2*^(2) = 16.58, *p* < .001, *V* = .10, small effect; Table 1). The standardized residuals indicated an overrepresentation of enrolled students with a non-Western migration background (*z*_res_ = 3.4) and an underrepresentation of enrolled students without a migration background (*z*_res_ = − 1.6). Participants and non-participants in PAP did not statistically significantly differ in terms of sex and parental education. However, compared to non-participants, participants in the PAP had a statistically significantly higher year 5 pu-GPA (*t* = 1.98, *df* = 831, *p* = .048, *d* = 0.20, small effect).

The number of selection candidates who took part in commercial coaching to prepare for medical school selection was remarkably small (*N* = 65; Table 1). The group of selection candidates who took part in commercial coaching did not differ statistically significantly in sex, parental education and year 5 pu-GPA from the group of selection candidates who did not engage in commercial coaching. However, a statistically significant difference between these groups was found for migration background (χ^*2*^(2) = 8.90, *p* = .012, *V* = 0.06, small effect), indicating an underrepresentation of students with a non-Western migration background (*z*_res_ = − 2.5) and an overrepresentation of selection candidates without a migration background (*z*_res_ = 1.6) in the group of commercial coaching participants.

### Research question 2: association between participation in preparatory activities and selection outcomes

Descriptive statistics of selection outcomes and early academic performance of participants versus non-participants of different preparatory activities are depicted in Additional file 2.

The results of the linear regression analyses indicated a statistically significant positive association between attending the Summer School and CV-scores (*B* = 0.79, 95% *CI* [0.69, 0.84], *p* < .001, adjusted *R*^*2*^ = .09, small effect; Table 2 - Model 1), as well as selection tests scores (*B* = 0.46, 95% *CI* [0.35, 0.56], *p* < .001, adjusted *R*^*2*^ = .03, small effect). This was also the case for the Coaching Day (CV-scores: *B* = 0.72, 95% *CI* [0.64, 0.79], *p* < .001, adjusted *R*^*2*^ = .13, medium effect; selection test scores: *B* = 0.45, 95% *CI* [0.37, 0.53], *p* < .001, adjusted *R*^*2*^ = .05, small effect). Although for commercial coaching the associations between participation and selection outcomes were also statistically significantly positive, the effects were negligible (CV-scores: *B* = 0.30, 95% *CI* [0.06, 0.54], *p* = .02, adjusted *R*^*2*^ = .002; selection test scores: *B* = 0.42, 95% *CI* [0.23, 0.70], *p* = .02, adjusted *R*^*2*^ = .005). Associations between participation in all three activities and both selection outcomes remained statistically significant after controlling for participation in other activities (Table 2 - Model 2), and for year 5 pu-GPA (Table 2 – Model 3).

**Table 2:** The association between selection outcomes and participation in each preparatory activity

			Z-score curriculum vitae			Z-score selection tests			Probability of enrolment	
Model		Participation in	*B*	95% *CI*	*R*^2^	*B*	95% *CI*	*R*^2^	*OR*	95% *CI*
1	A	Intercept	− 0.14	− 0.18, − 0.10	0.09	− 0.08	−0.13, − 0.04	0.03		
		Summer School (yes)	0.79***	0.69, 0.84		0.46***	0.35, 0.56		2.34	1.93, 2.85***
	B	Intercept	−0.40	−0.45, −0.34	0.13	−0.25	−0.31, − 0.19	0.05		
		Coaching Day (yes)	0.72***	0.64, 0.79		0.45***	0.37, 0.53		2.18	1.89, 2.52***
	C	Intercept	0.01	−0.04, 0.05	0.002	0.02	−0.02, 0.06	0.005		
		Commercial coaching (yes)	0.30*	0.06, 0.54		0.42**	0.18, 0.66		1.68	1.02, 2.78*
2		Intercept	−2.19***	−2,72, −1.66	0.17	−4.01***	− 4.55, − 3.46	0.06		
		Summer School (yes)	0.54***	0.44, 0.65		0.28***	0.17, 0.39		2.01	1.61, 2.51***
		Coaching Day (yes)	0.59***	0.51, 0.67		0.35***	0.27, 0.44		2.05	1.73, 2.43***
		Commercial coaching (yes)	0.38**	0.16, 0.61		0.47***	0.23, 0.70		1.94	1.16, 3.24*
3		Intercept	−2.19***	−2.74, −1.64	0.21	−3.99***	−4.61, −3.38	0.12		
		Summer School (yes)	0.48***	0.37, 0.58		0.24***	0.13, 0.35		2.56	1.97, 3.32***
		Coaching Day (yes)	0.52***	0.44, 0.60		0.30***	0.21, 0.38		2.34	1.93, 2.85***
		Commercial coaching (yes)	0.34**	0.13, 0.56		0.40***	0.18, 0.63		2.04	1.14, 3.67*
		Missing year 5 pu-GPA (yes)	−0.31***	−0.40, −0.22		−0.17***	−0.26, − 0.08		1.49	1.22, 1.80***
		Year 5 pu-GPA (continuous)	0.28***	0.20, 0.36		0.57***	0.48, 0.66		17.54	12.95, 23.74***

Pu-GPA = pre-university grade point average. Linear regression analyses were performed for the dependent variables Z-score on curriculum vitae and selection tests. Logistic regression analyses were performed for the dependent variable probability of enrolment. *B* refers to the unstandardized regression coefficient together with the 95% confidence interval (95% CI). OR refers to the odds ratio of the subgroups compared to the odds ratio of the reference group together with the 95% confidence interval (95% CI). All reported R^2^s are adjusted

*p < .05 **p < .01 ***p < .001

The results of the logistic regression analyses indicated a statistically significant positive association between participation in all preparatory activities and odds of enrolment. For Summer School, 66.9% of participating candidates were enrolled in the first year of medical school, while only 46.3% of the candidates who did not participate in Summer School were enrolled (*OR* = 2.34, 95% *CI* [1.93, 2.85], small effect; Table 2 – model 1). Similar effects were found for participation in Coaching Day (59.0% vs 39.8%; *OR* = 2.18, 95% *CI* [1.89, 2.52], small effect) and for participation in commercial coaching (58.0% versus 45.5%; *OR* = 1.68, 95% *CI* [1.02, 2.78], small effect). Associations between participation in all three activities and enrolment remained statistically significant after controlling for participation in other activities (Table 2 - Model 2), and for year 5 pu-GPA (Table 2 – Model 3).

**Fig. 3:**
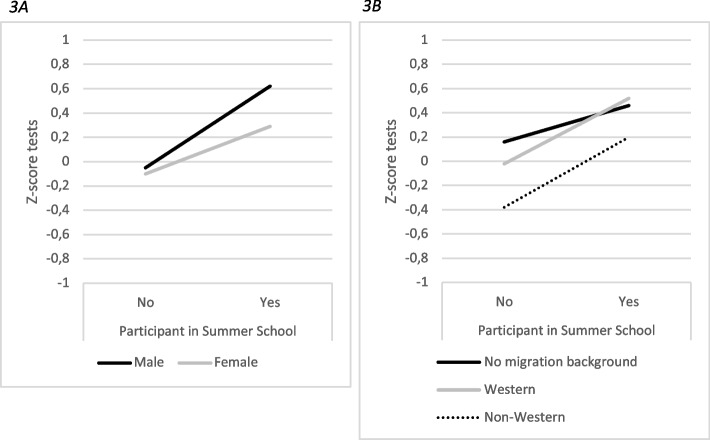
Statistically significant interaction effects between student characteristics and participation in Summer School for selection tests (*Z*-scores) Unadjusted mean *Z*-score on selection tests of participants versus non-participants in Summer School. Male: − 0.05 versus 0.62; female: − 0.1 versus 0.29 (3A); no migration background: 0.16 versus 0.46; Western: − 0.02 versus 0.52; non-Western: − 0.38 versus 0.20 (3B)

Subgroup analyses revealed that the association between participation in Summer School and selection test scores was stronger for males compared to females (*B* = 0.28, 95% *CI* [0.06, 0.50], *p* = .02; Additional file 3), and for Summer School participants with a non-Western migration background compared to those without a migration background (*B* = 0.26, 95% *CI* [0.03, 0.48], *p* = .03). For females, mean selection tests scores were 0.30 standard deviation (*SD*) higher for participants versus non-participants, while for participating males the mean selection tests scores were 0.67 *SD* higher (Fig. 3A).

Likewise, participating selection candidates with a non-Western migration background scored 0.58 *SD* higher than their non-participating counterparts, while there was only a difference of 0.30 *SD* between participants and non-participants for selection candidates without a migration background (Fig. 3B). Non-participating selection candidates with a non-Western migration background performed statistically significantly lower on selection tests (*B* = -0.47, 95% *CI* [− 0.56, − 0.37]), while the mean scores of participating selection candidates with a non-Western migration background were closer to the mean Z-score. An interaction effect in the same direction was observed between migration background (no migration background versus non-Western migration background) and CV-scores, although for this outcome measure the interaction effect was not statistically significant (*B* = 0.20, 95% *CI* [− 0.02, 0.42], *p* = .07). However, this interaction effect was not reflected by the probability of enrolment: even after controlling for participation, candidates with a non-Western migration background had statistically significantly lower chances of enrolment in the first year of medical school (Summer School: *OR* = 0.47, 95% *CI* [0.39, 0.60], small effect; Coaching Day: *OR* = 0.54, 95% *CI* [0.38, 0.75], small effect; Additional file 3), and the association between participation and odds of enrolment did not differ between applicants with a non-Western migration background and applicants without a migration background.

There was no statistically significant interaction effect between participation in Summer School and parental education. Additionally, no interaction effects were found between participation in Coaching Day and the different demographic variables.

### Research question 3: association between participation in preparatory activities and early academic performance

Regression analyses for each separate activity indicated only a positive association between participation in JMS and early academic performance, but not for the other activities (Table 3 – Model 1 A-E). The positive association for JMS remained after controlling for participation in PAP (Model 2B), but disappeared when controlling for year 6 pu-GPA (Model 3B).

**Table 3:** Results linear regression for association between early academic performance and participation in each preparatory activity

Model			*B*	95% *CI*	Adjusted *R*^2^
1	A	Intercept	6.29***	6.23, 6.35	0.006
		Participation in Summer School (yes)	-0.23**	−0.37, − 0.10	
	B	Intercept	6.44***	6.35, 6.53	0.02
		Participation in Coaching Day (yes)	−0.34***	− 0.45, − 0.23	
	C	Intercept	6.06***	5.99, 6.12	0.000
		Participation in commercial coaching (yes)	−0.11	− 0.46, 0.25	
	D	Intercept	6.18***	6.13, 6.24	0.21
		Participation in JMS (yes)	1.14***	0.88, 1.40	
	E	Intercept	6.22***	6.16, 6.28	0.000
		Participation in PAP (yes)	0.10	−0.06, 0.27	
2	A	Intercept	6.16***	6.04, 6.27	0.007
		Participation in Summer School (yes)	−0.12	− 0.26, 0.03	
		Participation in Coaching Day (yes)	−0.14*	− 0.28, − 0.00	
		Participation in commercial coaching (yes)	−0.14	− 0.50, 0.21	
		Participation in PAP (yes)	0.19*	0.01, 0.38	
	B	Intercept	6.17***	6.11, 6.23	0.21
		Participation in JMS (yes)	1.15***	0.89, 1.41	
		Participation in PAP (yes)	0.12	−0.04, 0.29	
3	A	Intercept	−1.52***	−2.29, − 0.75	0.26
		Participation in Summer School (yes)	−0.02	− 0.15, 0.11	
		Participation in Coaching Day (yes)	−0.00	−0.12, 0.12	
		Participation in commercial coaching (yes)	−0.09	−0.39, 0.22	
		Participation in PAP (yes)	0.12	−0.04, 0.28	
		Missing year 6 pu-GPA (yes)	−0.39**	−0.62, − 0.15	
		Year 6 pu-GPA (continuous)	1.06***	0.96, 1.17	
	B	Intercept	−2.09***	−2.68, −1.49	0.59
		Participation in JMS (yes)	0.04	−0.19, 0.27	
		Participation in PAP (yes)	0.11	−0.02, 0.25	
		Missing year 6 pu-GPA (yes)	−0.30**	−0.50, − 0.11	
		Year 6 pu-GPA (continuous)	1.14***	1.06, 1.22	

Dependent variable: weighted first course grade. *B* refers to the unstandardized regression coefficient together with the 95% confidence interval (95% CI)

*PAP* Pre-Academic Program, *pu-GPA* pre-university grade point average

* *p* < .05 ** *p* < .01 *** *p* < .001

When controlling for participation in other activities, the positive association between participation in PAP and first course grade became statistically significant, but the effect was negligible (*B* = 0.19, 95% *CI* [0.01, 0.38], *p* = .04, *R*^*2*^ = .007; Table 3 – Model 2A). Furthermore, this positive effect disappeared after controlling for year 6 pu-GPA (Table 3 – Model 3).

Without controlling for year 6 pu-GPA, participation in Summer School and Coaching Day were negatively associated with first course grade (respectively *B* = -0.23, 95% *CI* [− 0.37, − 0.10], *p* = .001, adjusted *R*^*2*^ = .006; *B* = -0.34, 95% *CI* [− 0.45, − 0.23], *p* < .001, adjusted *R*^*2*^ = 0.02, small effect; Table 3 – Model 1). This association diminished after controlling for participation in other activities (Table 3 – Model 2) and even disappeared after controlling for year 6 pu-GPA (Table 3 – Model 3). For enrolled students, year 6 pu-GPA of participants in Coaching Day and Summer School was indeed statistically significantly lower compared to that of non-participants (respectively: *M* = 7.18 vs. *M* = 7.46, *t*(1607) = 9.24, *p* < .001; *M* = 7.14 vs. *M* = 7.34, t(1607) = 6.06, *p* < .001; Additional file 1). This is caused by the fact that the analyses of year 6 pu-GPA also included students who were directly admitted based on a high pu-GPA.

Subgroup analyses revealed no statistically significant interaction effects in the association between participation in activities and early academic performance based on sex, migration background and parental education (Additional file 4).

## Discussion

The present study investigated the extent to which subgroups of applicants used free institutionally-provided preparatory activities for selection and transition into undergraduate medical school, as well as associations between usage and selection outcomes and early academic performance for subgroups of (prospective) students. The results indicate that free preparatory activities were generally equally used by subgroups of applicants regardless of ethnic and socioeconomic background. Nevertheless, the opportunity to participate in such free activities was taken less often by males compared to females. Commercial coaching activities were used less often applicants with a non-Western migration background. However, as very few applicants made use of commercial coaching and participation in free institutionally-provided preparatory activities was a stronger predictor of selection outcomes compared to participation in commercial coaching, commercial coaching was probably not a major threat to student diversity. Our findings furthermore suggest that participation in specific activities (e.g., Summer School) may be particularly beneficial for male applicants and applicants with a migration background in increasing selection outcomes. Additionally, findings regarding accessibility and effectiveness were comparable across free preparatory activities with differences in goals, timing and intensity. For instance, the Summer School was more intensive (5 days versus 1 day) and had a broader goal (studying medicine versus preparing for selection) than the Coaching Day, but associations between participation and selection outcomes were similar. Finally, the results indicate that after controlling for pu-GPA, participation in free preparatory activities was not positively associated with early academic performance.

The findings of the present study firstly indicate that free institutionally-provided preparatory activities were generally used to the same extent by applicants with different sociodemographic backgrounds, but were used less often by males. Although previous studies found that free preparatory activities were used less often by applicants with a lower SES [15, 16], this was not confirmed by the present study. Interestingly, the free activities provided in prior studies were focused on the preparation for a specific selection method (e.g., taking practice exam items), whereas the activities in the present study had more general aims (e.g., providing information about different aspects of the selection procedure). Although the type of information that was provided may play a role, further research is needed to determine why the threshold to participate was lower for applicants with lower SES in the present study. However, the discrepancy in findings may also be partly caused by the operationalization of SES. Prior research either used multiple categories [16], or multiple indicators [15] to operationalize SES. Although first-generation university status is a relevant variable in the context of transitioning into higher education [44], it may be the case that not all socioeconomic barriers to participate could be captured by this binary variable. This is supported by our finding that paid coaching activities were used less often by applicants with non-Western backgrounds, a factor associated with lower household income [45], but not by first-generation university applicants. This suggests that barriers to participate in preparatory activities are more related to finances than to previously suggested factors such as time and awareness [15]. Finally, the underrepresentation of males in preparatory activities may be related to gender differences in conscientiousness [46] and planning skills [47], which may be due to societal expectations. Female students in medicine have also been reported to be more intrinsically motivated, which is associated with improved self-regulatory behaviors [48, 49].

Secondly, our study adds to the literature by demonstrating associations between participating in free institutionally-provided preparatory activities and selection outcomes for different subgroups of applicants. Overall, participation in free preparatory activities was positively associated with selection outcomes, but this relationship was even stronger for applicants with a non-Western migration background and male applicants. Non-participating applicants with a migration background had poorer selection outcomes compared to their (participating and non-participating) traditional counterparts, which resonates with previous research [3, 5, 33], and Bourdieu’s notion on the reproduction of inequality through social and cultural practices that are associated with the dominant group [32]. A possible explanation for our results is that free institutionally-provided preparatory activities may counter this reproduction of inequality by reducing some of the aforementioned barriers [9, 11], and thereby increase the social and cultural capital of applicants with non-traditional backgrounds. Interestingly, the interaction effects between demographics and participation were only present for Summer School and not for the Coaching Day. This can also be related to Bourdieu’s theory [32]: since the Summer School was a more intensive program and was aimed at providing students a preview of studying medicine at university, it probably made students more familiar with the (implicit) rules and expectations of academic culture. However, a self-selection effect might also be present in this case. Given that the Summer School took place longer before the start of the selection procedure, students who were more motivated and better informed may have participated more often in this activity.

A third key finding is that participation in preparatory activities is not associated with early academic performance, and that pu-GPA can be a strong confounding factor in this type of intervention research. Without controlling for pu-GPA, participation in Summer School and Coaching Day were negatively associated with early academic performance, but this relationship disappeared after controlling for pu-GPA. This suggests that the lower first course grades of participants in the Summer School and Coaching Day were likely caused by existing group differences in, for instance, prior knowledge and study skills among enrolled students who participated. Participants in JMS and PAP, on the other hand, had considerably higher pu-GPAs compared to non-participants, and any positive associations between participation in these activities and early academic performance perished after controlling for pu-GPA. This is also a possible explanation for the fact that a previous study focusing on PAP did find more promising results with respect to predicting early academic performance, since that study did not control for pu-GPA [20]. Another possible explanation for this difference in findings is that contrary to the programs in the previous study, the medical program used selection, which can lead to higher overall academic performance [50], and potentially better prepared students. Nevertheless, as male students and students with sociodemographic minority backgrounds perform poorer in medical school [36, 37], the lack of interaction effects between participation and these variables suggests that the preparatory activities in their current form do not contribute to reducing this performance gap.

A strength of the present study is that we included a variety of activities, as well as multiple cohorts to strengthen our observations. One limitation is that only formal activities provided by either the university or commercial agencies were included. Informal activities, such as browsing the internet and consulting one’s network, may also contribute to selection outcomes. However, as previous research suggests that informal activities are less effective compared to formal activities [16], this probably did not distort our findings to a large extent. Another limitation relates to self-selection for participation in activities. Our results may be biased because of pre-existing differences between the groups of participants and non-participants [31]. For instance, applicants who decide to participate in preparatory activities may already be more motivated or more conscientious compared to non-participants. To mitigate this, we included pu-GPA as a control variable, as well as participation in other activities. However, a potential self-selection or ‘participation’ effect is not necessarily negative, since this has been linked to higher academic performance and higher retention [51]. Nevertheless, randomization of participants is needed to draw causal inferences.

Future research could investigate how preparatory activities can better meet the needs of male applicants and applicants with a non-Western background, because both groups are underrepresented in medical education [33]. With respect to males, research may want to focus on attraction, given their low application and participation rates. Students with a non-Western migration background, on the other hand, applied and participated relatively often, but had poorer academic performance. Research could therefore examine how preparatory activities and/or curricula could be adjusted to ensure their inclusion and retention in medical school. Furthermore, future studies could examine why certain preparatory activities seem to be particularly beneficial for male applicants and applicants with a non-Western migration background when it comes to improving selection outcomes. Since the aims of institutionally-provided preparatory activities go beyond academic success, future research can also include other outcome measures than academic performance, including sense of belonging, interaction with peers, self-efficacy and satisfaction with study choice [20].

From a practical stance, our results provide support for implementing free preparatory activities for undergraduate medical education. The high participation rates of free preparatory activities illustrate that the provision of such activities satisfies a need of prospective students. Moreover, it may reduce the unfair effects of commercial coaching. Since the usage of paid preparatory activities was smaller compared to previous studies [15, 17], and other Dutch medical programs [52], the provision of free preparatory activities is likely associated with a decreased demand for commercial coaching. Notwithstanding its limitations, the present study also suggests that selection outcomes, especially of underrepresented and non-traditional applicants, can be enhanced by providing relatively brief interventions. Simultaneously, the lack of association between participation and early academic performance suggests that support should not only be provided before entering the medical program, but that strategies to generate a diverse medical doctor population should be conceptualized in a broader attraction-selection-inclusion-retention framework [8], including efforts over the course of the educational program. This should also include exertions that move away from a deficit approach and towards inititatives that value the unique talents and potential of students with non-traditional backgrounds and draw upon their established capital [53, 54].

## Conclusions

In conclusion, the provision of free institutionally-provided preparatory activities can contribute to widening access in medical education in two ways: (1) by potentially improving the selection outcomes of applicants with non-traditional and underrepresented backgrounds, and (2) by reducing the consequences of unequal access to commercial coaching agencies. However, in order to further increase student diversity, adjustments to activities and/or curricula are required to ensure inclusion and retention in medical school.

## Supplementary Information

**Additional file 1.** Demographic characteristics of students enrolled in the first year of medical school.

**Additional file 2.** Descriptive statistics of selection outcomes and early academic performance of participants versus non-participants of preparatory activities.

**Additional file 3.** The association between selection outcomes and participation in each free institutionally provided preparatory activity for different subgroups

**Additional file 4.** Results of linear regression for the association between early academic performance and participation in each free institutionally provided preparatory activity for different subgroups

## Data Availability

The datasets generated and/or analyzed during the current study are not publicly available due to the sensitivity of the data, but are available from the corresponding author on reasonable request.

## Abbreviations

SES: Socioeconomic status
WA: Widening access
Pu-GPA: Pre-university grade point average
CV: Curriculum vitae
JMS: Junior Med School
PAP: Pre-Academic Program
CBS: Statistics Netherlands
