# Supplementary material for: The added value of free preparatory activities for widening access to medical education: a multi-cohort study
Source: BMC Med Educ. 2023 Mar 29;23:196. doi: 10.1186/s12909-023-04191-7 (PMC10053372; doi:10.1186/s12909-023-04191-7)
Supplement: Supplementary file 1 — Additional file 1. Demographic characteristics of students enrolled in the first year of medical school. [file 12909_2023_4191_MOESM1_ESM.pdf]

Additional file 1: Demographic characteristics of students enrolled in the first year of medical school

			Total	Sex		Migration background			Parental education[a]		Year 6 pu-GPA	
				Male	Female	No	Western	Non-western	No 1st gen	1st gen		
**Enrolled students**		*n*	1632	475	1157	1133	149	350	866	266		1613
		%		29.1	70.9	69.4	9.1	21.4	76.5	23.5		98.8
**Summer School participant**	Yes	*n*	369	94	275	264	25	80	224	67	*M*	7.14
		%	22.6	25.5	74.5	71.5	6.8	21.7	77.0	23.0	*SD*	0.54
	No	*n*	1263	381	882	869	124	270	642	119	*M*	7.34
		%	77.4	30.2	69.8	68.8	9.8	21.4	76.3	23.7	*SD*	0.62
**Coaching Day participant**	Yes	*n*	969	258	711	672	94	203	588	172	*M*	7.18
		%	59.4	26.6	73.3	69.3	9.7	20.9	77.4	22.6	*SD*	0.55
	No	*N*	663	217	446	461	55	147	278	94	*M*	7.46
		%	40.6	32.7	67.3	69.5	8.3	22.2	74.7	25.3	*SD*	0.65
**JMS participant**	Yes	*n*	76	25	51	52	6	18			*M*	8.23
		%	4.7	32.9	67.1	68.4	7.9	23.7			*SD*	0.50
	No	*n*	1556	450	1106	1081	143	332	886	266	*M*	7.25
		%	95.3	28.9	71.1	69.5	9.2	21.3	76.5	23.5	*SD*	0.58
**PAP participant**	Yes	*n*	217	60	157	128	16	73	108	43	*M*	7.31
		%	13.3	27.6	72.4	59.0	7.4	33.6	71.5	28.5	*SD*	0.58
	No	*n*	1415	415	1000	1005	133	277	758	223	*M*	7.29
		%	86.7	29.3	70.7	71.0	9.4	19.6	77.3	22.7	*SD*	0.61
**Commercial coaching participant**	Yes	*n*	38	8	30	26	5	7	27	11	*M*	7.10
		%	2.3	21.1	78.9	68.4	13.2	18.4	71.1	28.9	*SD*	0.52
	No	*n*	1094	307	787	766	97	231	839	255	*M*	7.14
		%	96.6	28.1	71.9	70.0	8.9	21.1	76.7	23.3	*SD*	0.53

Legend. JMS = Junior Med School; PAP = Pre-Academic Program; N = number of individuals; 1st-gen. = first-generation university student; Pu-GPA = pre-university grade point average; M = mean; SD = standard deviation. [a]parental education is known for less medical students, as this information was only available for candidates of the selection procedure who filled out the demographics survey
