# Supplementary material for: The added value of free preparatory activities for widening access to medical education: a multi-cohort study
Source: BMC Med Educ. 2023 Mar 29;23:196. doi: 10.1186/s12909-023-04191-7 (PMC10053372; doi:10.1186/s12909-023-04191-7)
Supplement: Supplementary file 2 — Additional file 2. Descriptive statistics of selection outcomes and early academic performance of participants versus non-participants of preparatory activities. [file 12909_2023_4191_MOESM2_ESM.pdf]

Additional file 2: Descriptive statistics of selection outcomes and early academic performance of participants versus non-participants of preparatory activities

Activity	Participant	Z-score curriculum vitae			Z-score selection tests			First course grade[a]		
		M	*SD*	95% *CI*	*M*	*SD*	95% *CI*	*M*	*SD*	95% *CI*
Summer School	Yes	0.64	0.89	0.56, 0.74	0.37	0.86	0.29, 0.46	5.95	1.06	5.83, 6.08
	No	-0.13	0.95	-0.17, 0.08	-0.04	0.99	-0.09, 0.00	6.09	1.08	6.01, 6.16
Coaching Day	Yes	0.32	0.96	0.27, 0.38	0.21	0.91	0.16, 0.26	6.00	1.05	5.93, 6.08
	No	-0.39	0.86	-0.44, -0.33	-0.20	1.02	-0.27, 0.14	6.16	1.13	6.04, 6.27
JMS	Yes							7.33	1.05	7.09, 7.56
	No							6.18	1.13	6.13, 6.24
PAP	Yes							6.20	1.04	6.03, 6.37
	No							6.03	1.08	5.96, 6.10
Commercial coaching	Yes	0.30	0.91	0.07, 0.52	0.44	0.85	0.23, 0.65	5.95	1.06	5.60, 6.30
	No	0.00	0.98	-0.04, .0.05	0.02	0.98	-0.02, 0.06	6.06	1.08	5.99, 6.12

Legend. PAP = Pre-Academic Program; M = mean; SD = standard deviation; 95% CI = 95% confidence interval. [a]range: 1-10, with 10 as the highest grade
