# Supplementary material for: The added value of free preparatory activities for widening access to medical education: a multi-cohort study
Source: BMC Med Educ. 2023 Mar 29;23:196. doi: 10.1186/s12909-023-04191-7 (PMC10053372; doi:10.1186/s12909-023-04191-7)
Supplement: Supplementary file 3 — Additional file 3. The association between selection outcomes and participation in each free institutionally provided preparatory activity for different subgroups [file 12909_2023_4191_MOESM3_ESM.pdf]

Additional file 3: The association between selection outcomes and participation in each free institutionally provided preparatory activity for different subgroups

	Z-score curriculum vitae				Z-score selection tests				Probability of enrolment			
	Summer School[a]		Coaching Day[b]		Summer School[a]		Coaching Day[b]		Summer School[a]		Coaching Day[b]	
	B	95% *CI*	*B*	95% *CI*	*B*	95% *CI*	*B*	95% *CI*	*OR*	95% *CI*	*OR*	95% *CI*
Intercept	-1.84***	-2.40, -1.28	-1.80***	-2.37, -1.24	-3.59***	-4.20, -2.98	-3.55***	-4.16, 2.93				
Participant (yes)	0.42***	0.28, 0.57	0.47***	0.35, 0.59	0.08	-0.07, 0.23	0.24***	0.11, 0.36	2.93***	2.00, 4.31	2.57***	1.93, 3.43
Sex (male)	-0.12**	-0.20, -0.03	-0.11	-0.23, 0.00	0.09	-0.00, 0.18	0.09	-0.03, 0.21	0.94	0.75, 1.17	0.93	0.69, 1.26
Migration background (Western)	-0.00	-0.14, 0.13	-0.12	-0.30, 0.06	-0.12	-0.26, 0.02	-0.18	-0.36, 0.01	0.73	0.52, 1.02	0.60*	0.38, 0.96
Migration background (non-Western)	-0.16**	-0.26, -0.07	-0.19**	-0.32, -0.06	-0.47***	-0.56, -0.37	-0.50***	-0.63, -0.37	0.47***	0.39, 0.60	0.54***	0.38, 0.75
Parental education (1st gen)	-0.14**	-0.23, -0.04	-0.12	-0.25, 0.00	-0.07	-0.16, 0.03	-0.03	-0.16, 0.10	0.93	0.73, 1.18	1.00	0.72, 1.39
Participant (yes) * Sex (male)	-0.04	-0.26, 0.17	-0.02	-0.18, 0.14	0.28*	0.06, 0.50	0.08	-0.09, 0.24	0.96	0.54, 1.69	0.98	0.65, 1.48
Participant (yes) * Migration background (Western)	-0.14	-0.54, 0.27	0.20	-0.05, 0.45	0.20	-0.23, 0.63	0.17	-0.09, 0.42	1.57	0.53, 4.61	1.58	0.83, 3.00
Participant (yes) * Migration background (non-Western)	0.20[+]	-0.02, 0.42	0.10	-0.07, 0.27	0.26*	0.03, 0.48	0.13	-0.04, 0.31	1.14	0.64, 2.02	0.84	0.54, 1.31
Participant (yes) * parental education (1st gen)	0.00	-0.22, 0.22	-0.03	-0.20, 0.14	-0.04	-0.27, 0.18	-0.09	-0.26, 0.08	0.51	0.2, 0.90	0.71	0.46, 1.09
Year 5 pu-GPA missing (yes)	-0.32***	-0.40, -0.23	-0.31***	-0.40, -0.23	-0.15**	-0.24, -0.06	-0.15**	-0.24, -0.06	1.49***	1.22, 1.82	1.49***	1.22, 1.82
Year 5 pu-GPA (continuous)	0.25***	0.17, 0.33	0.25***	0.17, 0.33	0.54***	0.45, 0.63	0.53***	0.44, 0.62	17.57***	12.90, 23.93	17.17***	12.62, 23.36
Adjusted R^2	0.22		0.22		0.17		0.17		n.a.		n.a.	

Legend. 1st gen = first-generation university student; pu-GPA = pre-university grade point average. Dependent variables: Z-score on curriculum vitae and selection tests. Linear regression analyses were performed for the dependent variables Z-score on curriculum vitae and selection tests. Logistic regression analyses were performed for the dependent variable probability of enrolment. B refers to the unstandardized regression coefficient together with the 95% confidence interval (95% CI). OR refers to the odds ratio of the subgroups compared to the odds ratio of the reference group together with the 95% confidence interval (95% CI). [a] adjusted for participation in Coaching Day and commercial coaching, and for pu-GPA. [b] adjusted for participation in Summer School and commercial coaching. [+]p=.07* p<.05 ** p<.01 *** p<.001
