# Supplementary material for: The added value of free preparatory activities for widening access to medical education: a multi-cohort study
Source: BMC Med Educ. 2023 Mar 29;23:196. doi: 10.1186/s12909-023-04191-7 (PMC10053372; doi:10.1186/s12909-023-04191-7)
Supplement: Supplementary file 4 — Additional file 4. Results of linear regression for the association between early academic performance and participation in each free institutionally provided preparatory activity for different subgroups [file 12909_2023_4191_MOESM4_ESM.pdf]

Additional file 4. Results of linear regression for the association between early academic performance and participation in each free institutionally provided preparatory activity for different subgroups

	Summer School[a]		Coaching Day[b]		JMS		PAP[c]	
	B	95% *CI*	*B*	95% *CI*	*B*	95% *CI*	*B*	95% *CI*
Intercept	-1.69***	-2.50, -0.89	-1.68***	-2.49, -0.88	-2.18***	-2.79, -1.57	-1.69***	-2.49, -0.89
Participant (yes)	-0.07	-0.25, 0.11	-0.01	-0.17, 0.16	0.07	-0.24, 0.38	0.09	-0.13, 0.31
Sex (male)	0.13	-0.01, 0.27	0.01	-0.19, 0.21	0.16**	0.06, 0.26	0.11	-0.02, 0.24
Migration background (Western)	0.28*	0.06, 0.50	0.50**	0.14, 0.85	0.14	-0.03, 0.31	0.26*	0.06, 0.47
Migration background (non-Western)	-0.09	-0.25, 0.08	-0.03	-0.27, 0,21	0.01	-0.11, 0.12	-0.06	-0.21, 0.10
Parental education (1st gen)	0.06	-0.10, 0.21	0.11	-0.11, 0.34			0.09	-0.06, 0.23
Participant (yes) * Sex (male)	0.03	-0.25, 0.32	0.20	-0.06, 0.45	-0.05	-0.52, 0.42	0.21	-0.17, 0.58
Participant (yes) * Migration background (Western)	-0.24	-0.73, 0.26	-0.38	-0.80, 0.04	-0.41	-1.23, 0.41	-0.30	-1.00, 0.40
Participant (yes) * Migration background (non-Western)	0.17	-0.14, 0.49	-0.03	-0.32, 0.26	-0.01	-0.52, 0.50	0.03	-0.36, 0.41
Participant (yes) * parental education (1st gen)	0.14	-0.16, 0.44	-0.04	-0.32, 0.23			-0.00	-0.38, 0.38
Year 6 pu-GPA missing (yes)	-0.42**	-0.66, -0.17	-0.44***	-0.68, -0.19	-0.33**	-0.53, -0.13	-0.41**	-0.65, -0.17
Year 6pu-GPA (continuous)	1.08***	0.97, 1.19	1.08***	0.97, 0.12	1.15***	1.06, 1.23	1.08***	0.97, 1.18
Adjusted R^2	0.27		0.27		0.59		0.27	

Legend. 1st gen = first-generation university student; pu-GPA = pre-university grade point average; PAP = Pre-Academic Program. Dependent variable: weighted first course grade. B refers to the unstandardized regression coefficient together with the 95% confidence interval (95% CI). [a] adjusted for participation in Coaching Day, PAP, and commercial coaching. [b] adjusted for participation in Summer School, PAP, and commercial coaching. [c] adjusted for participation in Summer School, Coaching Day, and commercial coaching. * p<.05 ** p<.01 *** p<.001
